# A Computer Color-Matching Study of Reverse Micellar Dyeing of Wool with Reactive Dyes

**DOI:** 10.3390/polym11010132

**Published:** 2019-01-14

**Authors:** Yanming Wang, Yiu-lun Tang, Cheng-hao Lee, Chi-Wai Kan

**Affiliations:** 1 Institute of Textiles and Clothing, The Hong Kong Polytechnic University, Hung Hom, Kowloon, Hong Kong, China; tcymwang@polyu.edu.hk (Y.W.); alan.yl.tang@connect.polyu.hk (Y.-l.T.); 2 Department of Applied Biology and Chemical Technology, The Hong Kong Polytechnic University, Hung Hom, Kowloon, Hong Kong, China; bcchlee@polyu.edu.hk

**Keywords:** wool fiber, non-ionic surfactant, octane, nonane, reverse micelle, reactive dye, color-matching, levelness

## Abstract

Computer color-matching (CCM) and the levelness of poly(ethylene glycol)-based reverse-micellar dyed wool fabrics in octane and nonane were investigated and compared with a conventional water-based dyeing system. Reflectance curves and calibration curves exhibited no chromatic change and maintained high linearity in both dyeing systems. The linearity of water-dyed calibration curves was slightly higher than that of the reverse-micellar dyed curves. The color yield, in term of K/S_sum_ values, of solvent-dyed samples was found to be generally higher than that of water-based dyed samples at various calibrated dye concentrations. The concentrations predicted by CCM were close to the theoretical concentrations for both dyeing methods. This indicates that octane- and nonane-assisted reverse-micellar dyeing of wool is able to generate color recipes comparable to the conventional water-based dyeing system. The solvent-dyed samples, measured by the relative unlevelness indices (RUI), exhibit good-to-excellent levelness, which is highly comparable with the water-dyed samples.

## 1. Introduction

Wool, with distinct characteristics of soft handle, good warmth retention, high moisture regain, and high ignition temperature, is an important natural animal protein fiber that helps human beings live an eco-friendly lifestyle [1,2]. Wool is an important fiber in the textile industry; however, due to the presence of a high number of disulfide cysteine cross-linkages (–S–S–), it has hydrophobicity on its surface [3,4] and, thus, problems of wettability and dyeability.

In the conventional water-based dyeing procedure, textile reactive dyes are widely used for coloration of wool fiber owing to their excellent fastness properties. Unlike the reactive coloration of cotton, the fixation of dye on wool fiber is achieved via an increase in temperature under weak acidic conditions (pH 4–6), instead of the addition of alkali, which may cause fiber degradation [5]. The dye-fiber interaction is relevant to the chemical bonding between water-soluble groups of the dyes and the amino and hydroxyl groups of wool fiber, improving the fixation rate and colorfastness [6]. However, the fixation of reactive dyes on wool is insufficient to achieve good wet-fastness properties since fixation via Coulombic interaction is unavoidable, and additional alkali after-treatment is required for neutralization [7,8]. In addition, the use of reactive dyes in the conventional water-based dyeing approach reveals some drawbacks, such as low dye fixation, requirement of a huge amount of dyeing auxiliaries, and high volume of effluent discharge [9]. These problems adversely affect the environment, as well as the quality of life of human beings, animals, and aquatic living beings, and it is contradictory to stringent environmental regulations.

To reduce wastewater discharges and environmental impacts, various methods were used to improve the exhaustion and fixation of dyes on wool fiber. These attempts included (a) pretreatment or modification of wool fiber before coloration [10,11,12,13,14,15,16]; (b) synthesis of novel dyestuffs [8,17]; (c) microencapsulation with liposomes [18,19]; (d) reuse of dyebath [20] and seawater [21]; (e) foam dyeing [22]; (g) ultrasound-assisted dyeing [23,24,25]; and (h) solvent-assisted dyeing using mixed solvent [26], supercritical critical fluid, and/or reverse micelle [27,28,29,30,31,32].

Reverse micelles are self-assembled colloidal structures formed by non-ionic surfactants in organic solvent with nanoscale water pools in hydrophilic cores [33]. Previous literature on the colorimetric measurement of textiles was achieved using spectrophotometry [34,35,36,37,38]. In our previous studies, we successfully used poly(ethylene glycol) (PEG)-based non-ionic surfactants to facilitate the formation of reverse micelles, the analysis of stability and dispersity of well-defined reverse micelles with different reverse-micellar dyeing parameters, and the application of the computer color-matching (CCM) technique to study the dyeing properties of cotton fiber in different non-aqueous solvent media [39,40,41,42,43,44]. As the aesthetic appearance of most textile products is a mixture of colors rather than a single color, CCM is an important aspect that cannot be neglected in industrial dyeing applications [45,46]. However, to the best of our knowledge, most researchers focused on dyeing of wool with a single color, while the feasibility of CCM’s application on wool fiber is still unknown and is yet to be reported in the literature. 

The main aims of this study included (a) construction of calibration curves for reactive dyes in conventional aqueous and alkane non-aqueous dyeing media; (b) simulated dyeing of wool fabrics with known dye concentration for both dyeing approaches; (c) measurement of the reflectance, K/Ssum, CIE L*a*b* values, and levelness of the dyed wool samples; (d) prediction of the color recipe between batch samples and standard samples using computer color-matching (CCM); and (e) assessment of the difference between computer color-matching and levelness of conventional water-dyed samples and alkane-dyed samples.

## 2. Materials and Methods

### 2.1. Materials and Reagents

Woven wool plain fabrics (73 warps per inch × 60 wefts per inch) were firstly immersed in acetone (GR grade, Duksan, Gyunggido, Korea) for 5 min and then rinsed with 2 g/L soap. After soaping, the fabrics were washed in cold water and then air-dried at room temperature. The air-dried fabrics were then conditioned for at least 24 h at 20 ± 2 °C and relative humidity of 65 ± 2% prior to further experiment. The non-ionic surfactant, poly(ethylene glycol) (12) tridecylether (C_13_H_27_(OCH_2_CH_2_)_12_OH) was used (reagent grade, Sigma Aldrich, St. Louis, MO, USA). Octane and nonane (reagent grade, ACROS, Fair Lawn, NJ, USA) were used as the dyeing media and *n*-octanol (reagent grade, Alfa Aesar, Heysham, UK) was used as a co-surfactant in the dyeing process. Acetic acid (reagent grade, Sigma Aldrich, St. Louis, MO, USA) and sodium sulfate (reagent grade, Sigma Aldrich, St. Louis, MO, USA) were used in conventional aqueous dyeing. Realan reactive dyes (Realan Red EHF, Realan Yellow EHF, and Realan Blue EHF, Dystar, Shanghai, China) were used directly without further purification.

### 2.2. Preparation of Calibration Curvex

**Figure 1:**
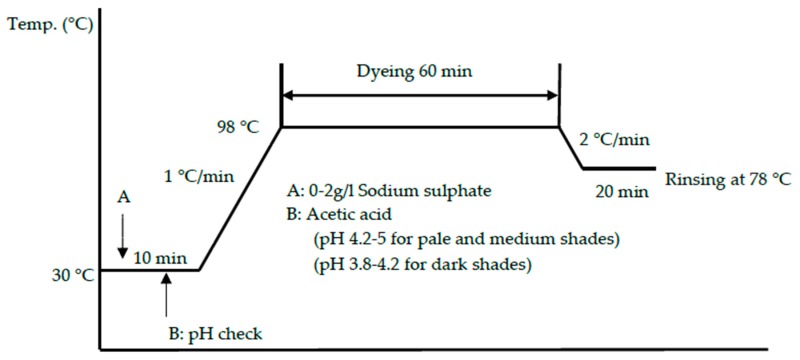
Profile of the conventional dyeing approach with salt.

**Figure 2:**
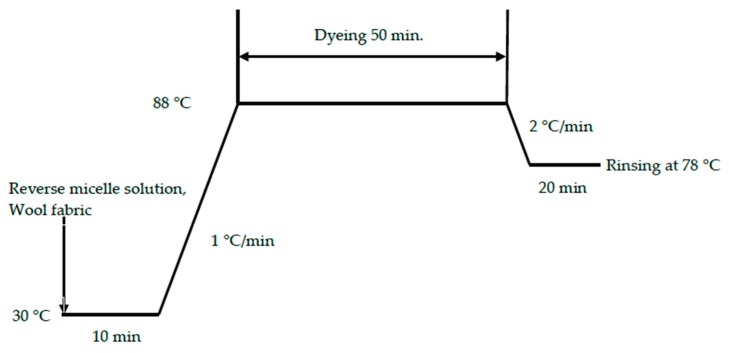
Profile of the reverse-micelle dyeing approach in octane and nonane without salt.

Wool woven fabrics were used. Calibrated dyeing of reverse-micellar dyeing and conventional aqueous dyeing (dye concentrations of 0.1%, 0.5%, 1.5%, 2.5%, and 3.5% of weight of wool fiber (owf)) was conducted with dyeing parameters shown in Table 1 and Table 2, respectively. Figure 1 and Figure 2 show the dyeing profiles of the conventional aqueous dyeing approach and the alkane reverse-micellar dyeing approach, respectively. The dyed samples used for the preparation of calibration curves were named as batch samples.

### 2.3. Simulated Dyeing

Simulated dyeing using known concentrations of dye was launched to predict the dye concentrations of calibrated conventional water-based and reverse-micellar methods. The dyed woven wool fabrics were used for color-matching and these samples were named as standard samples. Table 3 shows the concentrations of dye used for the preparation of color mixtures.

### 2.4. Plot of Calibration Curves

A spectrophotometer (Color Eye 7000A, X-Rite, Grand Rapids, MI, USA) was used for measuring the color yield of the dyed samples. The K/S_sum_ value was obtained by summation of K/S values within 400–700-nm wavelength range. The condition of measurement on the dyed fabric surface was specular reflection under a large aperture with a diameter of 30 mm. A 10° observer angle and a D_65_ light source were used. Dyed samples were folded to ensure opacity. Graphical plots (calibration curves) of K/S_sum_ value versus concentration of dye (%) were then prepared.

The color yield, K/S value, was calculated from Equation (1) at wavelengths ranging from 400 to 700 nm in steps of 10 nm. As the K/S value increases, the dye uptake and color yield improve.

K/S = (1 − R)^2^/2R, (1)

where K is the absorption coefficient, depending on the colorant concentration, S is the scattering coefficient, caused by the dyed substrate, and R is the reflectance factor of the colored sample at a specific wavelength [39].

### 2.5. Dye Recipe Prediction

Nine color difference equations, including CIE L*a*b*, CIE L*u*v*, ANLAB, Hunter lab, FMC2, JPC 79, CMC 1.0, BFD 1.0, and CIE94 1.0, were used to predict the dye recipe. Color yields of samples dyed with different concentrations of dye were measured with apparatus and conditions similar to those mentioned in Section 2.4. 

### 2.6. The CIE L*a*b* Value Measurement

CIE L*a*b* values of the dyed fabrics were measured with similar apparatus and conditions as those mentioned in Section 2.4.

### 2.7. Levelness Measurement

Relative unlevelness indices (RUI), an indicator of levelness of the dyed samples, were obtained by calculating the reflectance values of three randomly selected locations of standard and batch samples with the use of Equations (2)–(5) [47]. The interpretation of the value of RUI is as listed in Table 4.

(2) $$s_{\lambda }=\sqrt{\frac{\sum_{i=1}^{n}{\left(R_{i}-\bar{R}\right)}^{2}}{n-1}};$$

(3) $$\left({\mathrm{RUI}}_{u}\right)=\;\overset{700}{\underset{\lambda =400}{\sum }}s_{\lambda };$$

(4) $$\left({\mathrm{RUI}}_{c}\right)=\;\overset{700}{\underset{\lambda =400}{\sum }}s_{\lambda }/\bar{R};$$

(5) $$\mathrm{RUI}=\;\overset{700}{\underset{\lambda =400}{\sum }}(s_{\lambda }/\bar{R\;}){\;V}_{\lambda }.$$

## 3. Results

### 3.1. Reflectance Values of the Dyed Samples

**Figure 3:**
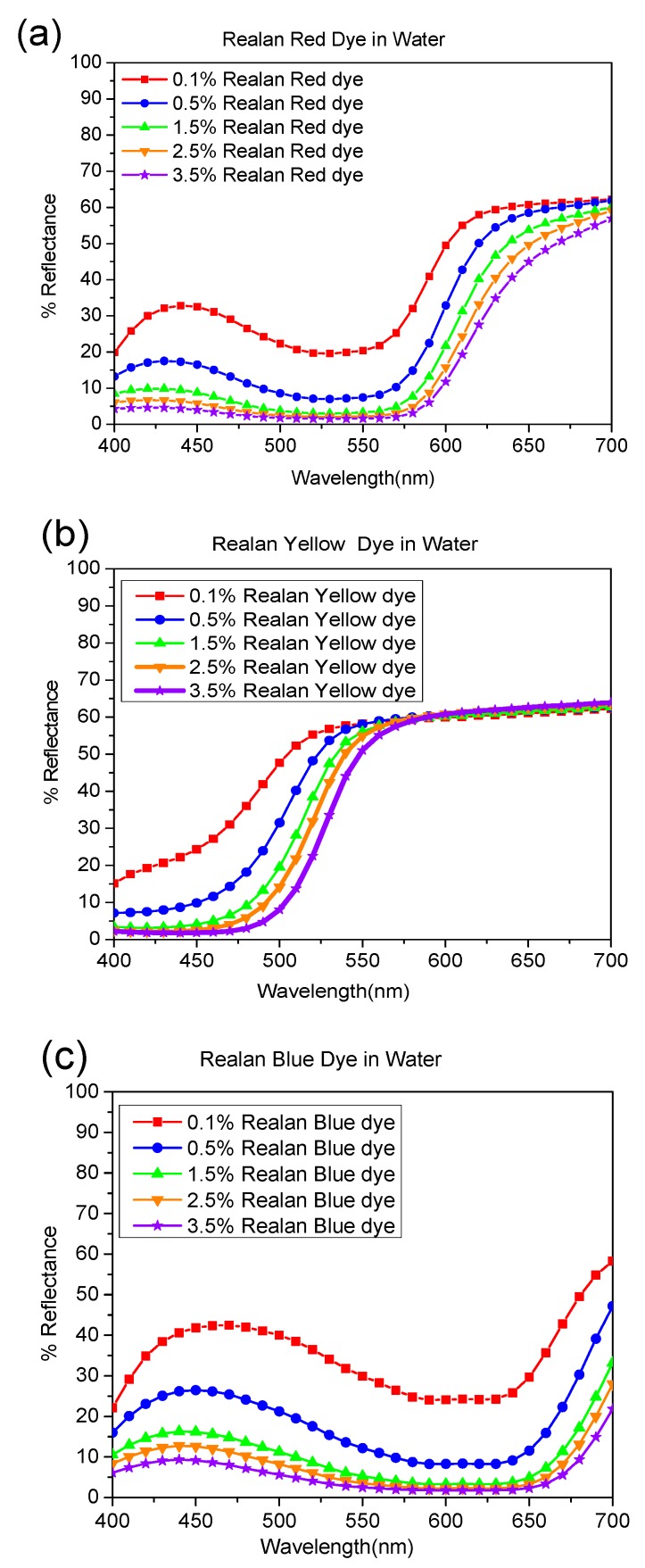
Percentage of reflectance value vs. wavelength (nm) of wool fibers dyed by (**a**) Realan Reactive Red, (**b**) Realan Reactive Yellow, and (**c**) Realan Reactive Blue in water.

**Figure 4:**
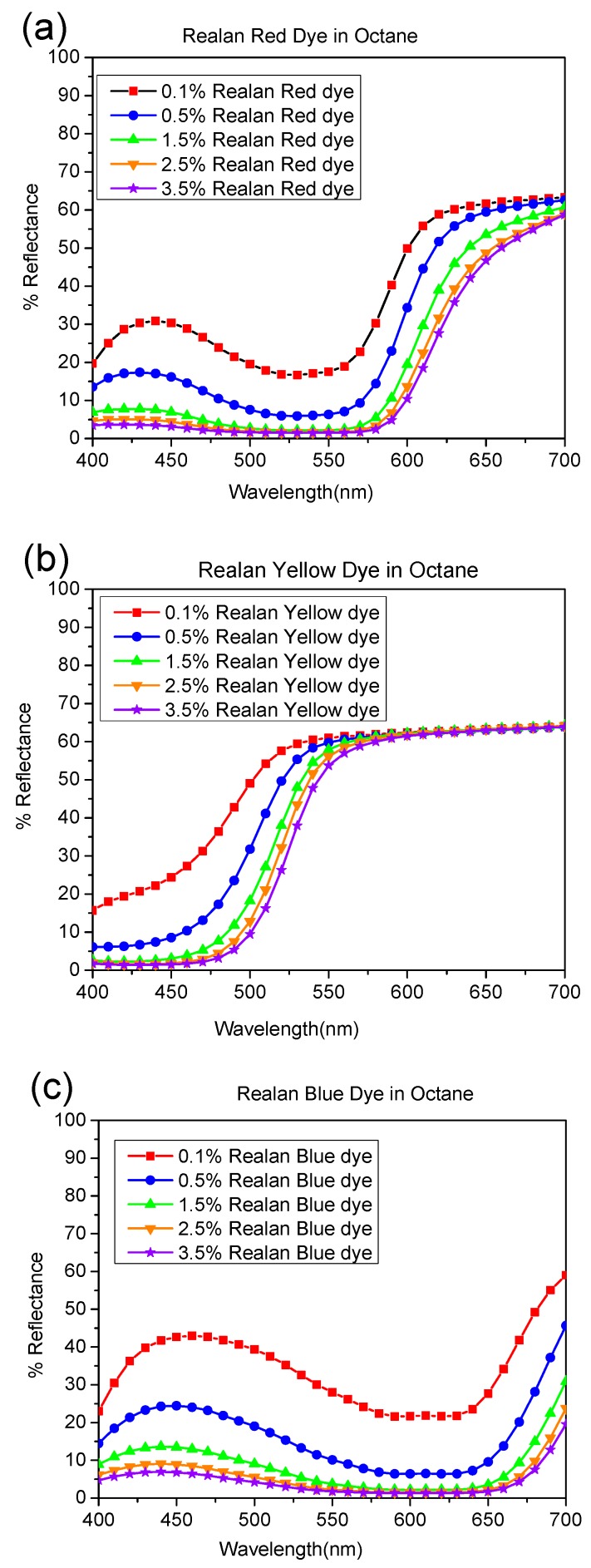
Percentage of reflectance value vs. wavelength (nm) of wool fibers dyed by (**a**) Realan Reactive Red, (**b**) Realan Reactive Blue, and (**c**) Realan Reactive Yellow in octane.

**Figure 5:**
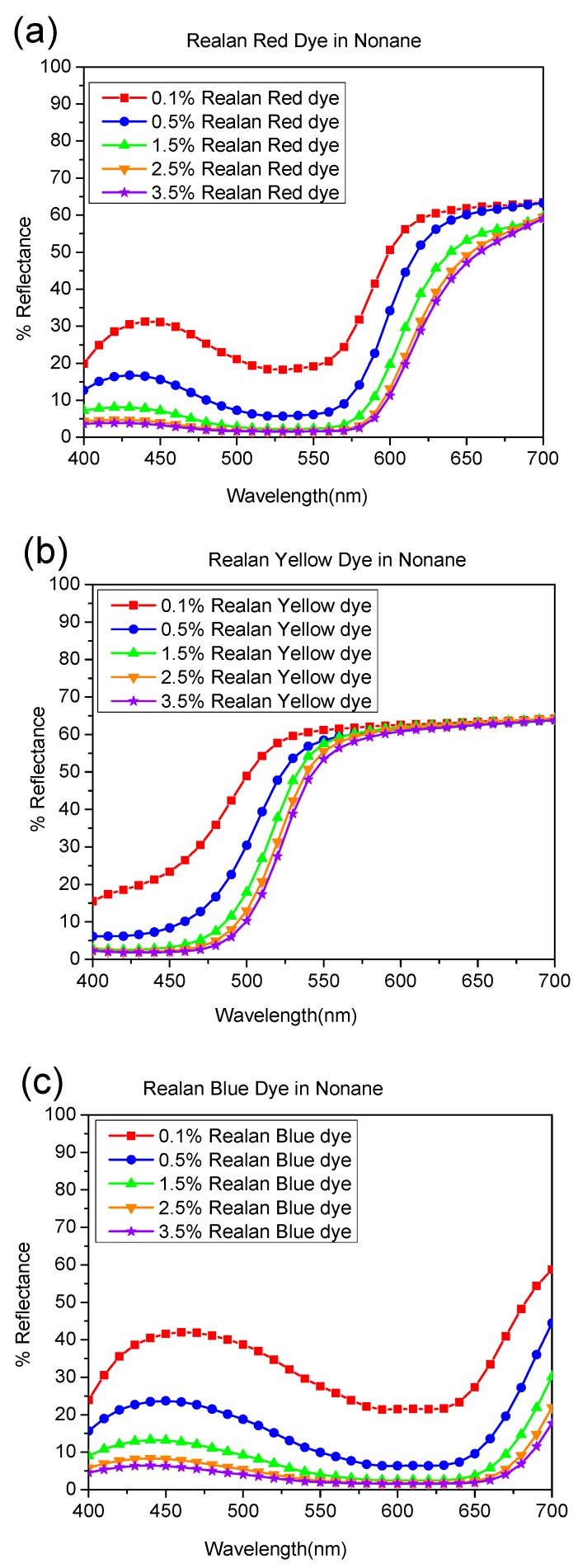
Percentage of reflectance value vs. wavelength (nm) of wool fibers dyed by (**a**) Realan Reactive Red, (**b**) Realan Reactive Yellow, and (**c**) Realan Reactive Blue in nonane.

The reflectance curves for a set of wool fabrics dyed with reactive dyes (red, yellow, and blue) in aqueous, octane, and nonane media are presented in Figure 3, Figure 4 and Figure 5, respectively. The shapes of the reflectance curves for the set of primary dyes should be almost similar (identical), without crossed lines.

As indicated by Figure 3, Figure 4 and Figure 5, the reflectance curves of fabrics dyed in higher concentrations are presented at the bottom of the graph with lower reflectance values, which indicates that more dyes were absorbed and bonded to fabrics, resulting in darker shades, and vice versa. From the evaluation of reflectance-versus-wavelength graphs, reflectance curves of the set of primary dyes in all concentrations revealed their own color patterns, which were almost identical in shape, and the results obtained were highly consistent.

**Figure 6:**
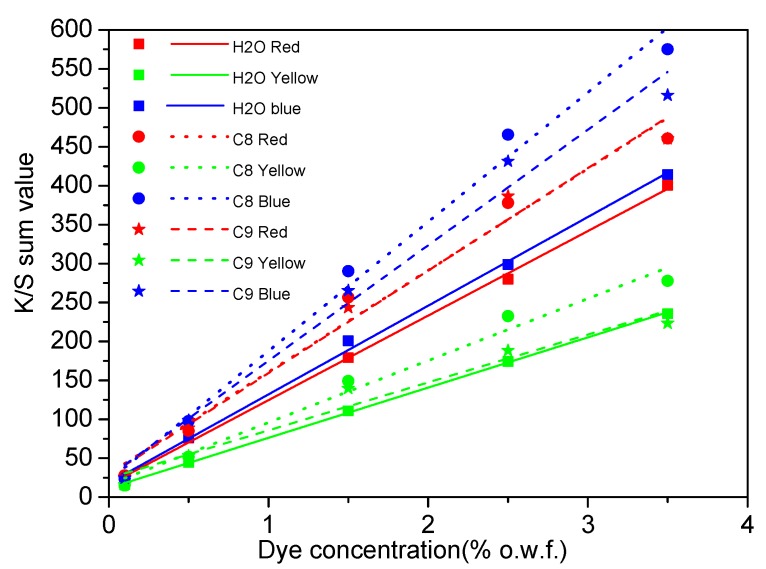
Calibration curves of dyed batch samples in water, octane, and nonane for color-matching.

Moreover, there were curves with a trough profile in the reflectance spectra. These trough profiles became deeper when dye concentration increased, since more light was selectively absorbed by the dye molecules. For instance, as shown in Figure 3a, Figure 4a, Figure 5a, and Figure 6a, the dyed wool fabric appeared “red” since violet-to-yellow light (400–590 nm) was absorbed, leaving predominantly orange-to-red light (590–725 nm) in the reflected beam. The increase in spectral reflectance with decreasing dye concentration occurred since the dye on the wool surface scattered light more effectively, resulting in a greater portion of the light beam being reflected back from the dyed wool.

As illustrated in Figure 3, Figure 4 and Figure 5, reflectance curves of the water-based and reverse-micellar dyed wool maintained identical profiles and exhibited no substantial peak shift in terms of wavelength. This indicates that the reactive dyes remained highly stable in terms of chemical structure after encapsulation in the reverse micelles and complete dyeing process in alkane solvent medium.

### 3.2. CIE L*a*b* Values

Table 5 depicts CIE L*a*b* values of wool fabrics dyed in water, octane, and nonane dyeing media. With regards to the red color, wool fabrics dyed using the reverse-micellar dyeing approach in octane and nonane solvent generally had lower L*, and higher a* and b* values than those seen using the conventional water-based dyeing approach. The measured CIE L*a*b* values indicate that alkane solvent-dyed fabrics in red are darker, redder, and yellower than the conventional water-dyed wool fabrics. Concerning the yellow color, wool fabrics dyed using the reverse-micellar dyeing approach in octane and nonane solvent generally had higher L*, lower a*, and higher b* values than those seen in the conventional water-based dyeing approach. This means reverse-micellar dyed fabrics in yellow color are lighter, greener, and yellower than the conventional water-dyed wool fabrics. Regarding the blue color, wool woven fabrics dyed in octane and nonane solvent generally had lower L*, higher a*, and lower b* values than fabrics dyed in water, indicating that the resultant blue color of reverse-micelle dyeing is darker, redder, and bluer than that of the conventional water-based dyeing.

### 3.3. Computer Color-Matching

#### 3.3.1. Linearity of the Calibration Curves

Calibration curves of the red, yellow, and blue batch samples dyed in water and reverse-micellar octane and nonane media are illustrated in Figure 6. The K/S_sum_ values of red, yellow, and blue samples dyed in the reverse-micellar system were higher than those in water system, indicating that the octane and nonane dyeing system can obtain better color yield than the water-dyeing system.

As illustrated in Figure 6 and Table 6, values of *R*^2^ of water-dyed fabrics ranged from 0.997 (water blue) to 0.999 (water yellow), whereas *R*^2^ values of octane-dyed and nonane-dyed fabrics ranged from 0.976 (octane red) to 0.987 (octane blue), and from 0.952 (nonane yellow) to 0.980 (nonane blue), respectively. This indicates that the calibration curves of both reverse-micellar dyed and water-dyed samples are linear-type functions and, thus, appropriate for computer color-matching.

#### 3.3.2. CCM Results

The color-matching predictions of water-dyed standard wool samples with several color difference formulae are presented in Table 7. The generated color-matching recipes of Sample 1 were nearly the same (red: 0.108; yellow: 0.114; blue: 0.095) as each other when different formulae were employed. Consistent results were also obtained for color-matching recipes of Sample 2 (red: 0.477; yellow: 0.488; blue: 0.554) and Sample 3 (red: 1.021; yellow: 0.974; blue: 1.081) through different formulations. However, it was observed that variations between color-matching recipes and samples dyed in 1.5% and 3.0% concentrations were generally larger than seen in the case of recipes with 0.3% dye concentration.

The color-matching recipes of octane-dyed standard wool samples are shown in Table 8. Results were found to be consistent for color-matching predictions of Sample 4 (red: 0.080; yellow: 0.113; blue: 0.105), Sample 5 (red: 0.543; yellow: 0.439; blue: 0.459), and Sample 6 (red: 1.144; yellow: 0.971; blue: 0.959), even though different formulae were used.

The color-matching recipes of nonane-dyed standard wool samples are presented in Table 9. The color-matching predictions of Sample 7, 8, and 9 were (red: 0.086; yellow: 0.081; blue: 0.097), (red: 0.530; yellow: 0.428; blue: 0.464), and (red: 1.107; yellow: 0.960; blue: 0.952), respectively.

Table 10 shows the percentage difference (%) between the theoretical and measured dye concentrations used for standard wool samples dyed by water, octane reverse-micellar, and nonane reverse-micellar approaches using various color difference formulae. Measured values below the expected concentrations were due to the fact that dye molecules were non-uniformly distributed on fabric matrices. Measured values above the predicted concentration were due to the occurrence of various sizes of dye agglomerates, as well as interfering incident light absorption and scattering. In addition, the variation between theoretical and measured concentration was influenced by the linearity of the calibration curves. Generally speaking, a higher linearity of the calibration curves may increase the accuracy and reproducibility of the result, thus leading to a smaller difference between the theoretical and measured concentrations.

### 3.4. Levelness

The relative unlevelness indices (RUI) and visual levelness assessments of water-dyed, octane-dyed, and nonane-dyed wool fabrics are illustrated in Table 11. Water-dyed fabrics had values between 0.05 and 0.46. Octane-dyed and nonane-dyed wool fabrics had values between 0.04 and 0.48 and between 0.04 and 0.43, respectively. The results indicate that samples dyed by water and solvent methods resulted in good-to-excellent levelness. 

## 4. Conclusions

Computer color-matching and the levelness of octane and nonane reverse-micellar dyed wool fabrics were investigated using PEG-based non-ionic surfactants, and the results were found comparable to those found for the conventional water-based dyeing system. No chromatic change was observed from the measured reflectance values. The calibration curves were almost linear in reflectance functions for both dyeing systems. The linearity of the water-dyed calibration curves was slightly higher than that for the reverse-micellar dyed curves. However, the color yield in terms of K/S_sum_ values for solvent-dyed samples was found to be higher than that for the water-dyed samples at each calibrated dye concentration. CCM was conducted using various spectral matching methods, and the results revealed that the CCM-predicted concentrations closely matched with the theoretical concentrations for both methods. This indicates that the octane and nonane dyeing of wool can achieve color-matching comparable to the water dyeing of wool. The RUI results implied that both water-dyed and solvent-dyed wool samples can subjectively and objectively achieve good-to-excellent levelness performance.

## Figures and Tables

**Table 1:** Dyeing parameters for octane and nonane.

Parameter	Value
Wool-to-solvent weight ratio (*w*/*v*)	1:10
Surfactant-to-co-surfactant molar ratio	1:8
Surfactant-to-water molar ratio	0.04:1
Water-pool volume for dye (mL)	0.5
Dyeing time (min)	50
Dyeing temperature (°C)	88

**Table 2:** The concentration of Na_2_SO_4_ and pH at various weight percentages of reactive dye for conventional water dyeing.

Liquid Ratio 50:1, 98 °C						
Dye	% of weight of wool fiber (% owf)	0.1	0.5	1.5	2.5	3.5
Salt (Na_2_SO_4_)	g/L	2	2	2	2	2
pH		5	4.5	4.2	4	3.8

**Table 3:** Dye concentrations (%) for preparing color mixtures.

Solvent	Standard Sample	Red (%)	Yellow (%)	Blue (%)
Water	Sample 1	0.100	0.100	0.100
	Sample 2	0.500	0.500	0.500
	Sample 3	1.000	1.000	1.000
Octane	Sample 4	0.100	0.100	0.100
	Sample 5	0.500	0.500	0.500
	Sample 6	1.000	1.000	1.000
Nonane	Sample 7	0.100	0.100	0.100
	Sample 8	0.500	0.500	0.500
	Sample 9	1.000	1.000	1.000

**Table 4:** Relative unlevelness index (RUI) interpretation [41].

Visual Appearance of Levelness	RUI
Excellent (unlevelness not detectable)	<0.2
Good (noticeable unlevelness under close examination)	0.2–0.49
Poor (apparent unlevelness)	0.5–1.0
Bad (conspicuous unlevelness)	>1.0

**Table 5:** The CIE L*a*b* values of dyed wool fabrics in various dyeing media (woven wool fabrics, Realan reactive dye).

Solvent	Water			Octane			Nonane		
Sample (%)	L*	a*	b*	L*	a*	b*	L*	a*	b*
Red 0.1	61.337	31.254	−1.043	59.61	35.431	−0.864	60.768	33.315	0.030
Red 0.5	47.070	45.574	1.972	46.484	48.976	1.939	46.213	49.451	2.772
Red 1.5	38.009	49.965	6.687	35.454	52.490	9.185	35.691	51.966	8.551
Red 2.5	33.238	49.285	9.580	31.108	50.184	12.679	33.507	53.042	16.384
Red 3.5	29.518	47.711	12.063	28.775	48.130	15.538	28.382	47.09	15.652
Yellow 0.1	79.059	-5.516	35.130	80.338	−5.813	36.958	80.371	−6.052	38.364
Yellow 0.5	76.850	-2.476	61.000	77.565	−2.974	65.359	76.918	−1.931	65.065
Yellow 1.5	74.322	3.117	78.398	74.937	3.941	84.543	74.800	4.522	84.212
Yellow 2.5	73.081	7.676	86.107	73.450	7.847	91.897	73.174	8.393	88.616
Yellow 3.5	70.665	14.771	90.549	71.927	11.824	93.782	71.920	11.288	90.928
Blue 0.1	62.619	−7.199	−12.262	61.184	−7.351	−15.578	60.811	−7.413	−15.078
Blue 0.5	43.906	−5.392	−22.837	40.789	−5.312	−24.752	40.515	−5.380	−24.244
Blue 1.5	30.788	−2.005	−25.929	26.587	−1.112	−26.841	27.289	−1.869	−24.966
Blue 2.5	25.727	−0.193	−25.902	20.334	1.545	−24.503	20.67	0.629	−21.704
Blue 3.5	21.181	1.884	−23.685	17.507	2.150	−21.859	18.066	1.871	−19.012

**Table 6:** *R*^2^ values of dyeing in three primary colors based on dyeing method.

Dyeing Medium	*R*^2^ Value
Water (red)	0.998
Water (yellow)	0.999
Water (blue)	0.997
Octane (red)	0.976
Octane (yellow)	0.977
Octane (blue)	0.987
Nonane (red)	0.978
Nonane (yellow)	0.952
Nonane (blue)	0.980

**Table 7:** Color-matching recipes of water-dyed standard wool samples.

Formulae	Color	Water-Based Wool Dyeing		
		Sample 1 (0.3%)	Sample 2 (1.5%)	Sample 3 (3%)
Theoretical	Red	0.100	0.500	1.000
	Yellow	0.100	0.500	1.000
	Blue	0.100	0.500	1.000
CIE L*a*b*	Red	0.108	0.477	1.021
	Yellow	0.114	0.488	0.974
	Blue	0.095	0.554	1.081
CIE L*u*v*	Red	0.108	0.477	1.021
	Yellow	0.114	0.488	0.974
	Blue	0.095	0.554	1.081
ANLAB	Red	0.108	0.477	1.021
	Yellow	0.114	0.488	0.974
	Blue	0.095	0.554	1.081
Hunter lab	Red	0.108	0.477	1.021
	Yellow	0.114	0.488	0.974
	Blue	0.095	0.554	1.081
FMC2	Red	0.108	0.477	1.021
	Yellow	0.114	0.488	0.974
	Blue	0.095	0.554	1.081
JPC79	Red	0.108	0.477	1.021
	Yellow	0.114	0.488	0.974
	Blue	0.095	0.554	1.081
CMC 1.0	Red	0.108	0.477	1.021
	Yellow	0.114	0.488	0.974
	Blue	0.095	0.554	1.081
BFD 1.0	Red	0.108	0.477	1.021
	Yellow	0.114	0.488	0.974
	Blue	0.095	0.554	1.081
CIE94 1.0	Red	0.108	0.477	1.021
	Yellow	0.114	0.488	0.974
	Blue	0.095	0.554	1.081

**Table 8:** Color-matching recipes of octane-dyed standard wool samples.

Formulae	Color	Octane Wool Dyeing		
		Sample 4 (0.3%)	Sample 5 (1.5%)	Sample 6 (3%)
Theoretical	Red	0.100	0.500	1.000
	Yellow	0.100	0.500	1.000
	Blue	0.100	0.500	1.000
CIE L*a*b*	Red	0.080	0.543	1.144
	Yellow	0.113	0.439	0.971
	Blue	0.105	0.459	0.959
CIE L*u*v*	Red	0.080	0.543	1.144
	Yellow	0.113	0.439	0.971
	Blue	0.105	0.459	0.959
ANLAB	Red	0.080	0.543	1.144
	Yellow	0.113	0.439	0.971
	Blue	0.105	0.459	0.959
Hunter lab	Red	0.080	0.543	1.144
	Yellow	0.113	0.439	0.971
	Blue	0.105	0.459	0.959
FMC2	Red	0.080	0.543	1.144
	Yellow	0.113	0.439	0.971
	Blue	0.105	0.459	0.959
JPC79	Red	0.080	0.543	1.144
	Yellow	0.113	0.439	0.971
	Blue	0.105	0.459	0.959
CMC 1.0	Red	0.080	0.543	1.144
	Yellow	0.113	0.439	0.971
	Blue	0.105	0.459	0.959
BFD 1.0	Red	0.080	0.543	1.144
	Yellow	0.113	0.439	0.971
	Blue	0.105	0.459	0.959
CIE94 1.0	Red	0.080	0.543	1.144
	Yellow	0.113	0.439	0.971
	Blue	0.105	0.459	0.959

**Table 9:** Color-matching recipes of nonane-dyed standard wool samples.

Formulae	Color	Nonane Solvent-Assisted Dyeing		
		Sample 7 (0.3%)	Sample 8 (1.5%)	Sample 9 (3%)
Theoretical	Red	0.100	0.500	1.000
	Yellow	0.100	0.500	1.000
	Blue	0.100	0.500	1.000
CIE L*a*b*	Red	0.086	0.530	1.107
	Yellow	0.081	0.428	0.960
	Blue	0.097	0.464	0.952
CIE L*u*v*	Red	0.086	0.530	1.107
	Yellow	0.081	0.428	0.960
	Blue	0.097	0.464	0.952
ANLAB	Red	0.086	0.530	1.107
	Yellow	0.081	0.428	0.960
	Blue	0.097	0.464	0.952
Hunter lab	Red	0.086	0.530	1.107
	Yellow	0.081	0.428	0.960
	Blue	0.097	0.464	0.952
FMC2	Red	0.086	0.530	1.107
	Yellow	0.081	0.428	0.960
	Blue	0.097	0.464	0.952
JPC79	Red	0.086	0.530	1.107
	Yellow	0.081	0.428	0.960
	Blue	0.097	0.464	0.952
CMC 1.0	Red	0.086	0.530	1.107
	Yellow	0.081	0.428	0.960
	Blue	0.097	0.464	0.952
BFD 1.0	Red	0.086	0.530	1.107
	Yellow	0.081	0.428	0.960
	Blue	0.097	0.464	0.952
CIE94 1.0	Red	0.086	0.530	1.107
	Yellow	0.081	0.428	0.960
	Blue	0.097	0.464	0.952

**Table 10:** Percentage (%) difference of dyed standard wool samples.

		Percentage Difference (%)								
Formulae	Color	Water-Based Dyeing (%)			Octane Dyeing (%)			Nonane Dyeing (%)		
		Sample1 (0.3%)	Sample2 (1.5%)	Sample3 (3%)	Sample4 (0.3%)	Sample5 (1.5%)	Sample6 (3%)	Sample7 (0.3%)	Sample8 (1.5%)	Sample9 (3%)
CIE L*a*b*	Red	↑8.00	↓4.60	↑2.10	↓20.00	↑8.60	↑14.40	↓14.00	↑6.00	↑10.70
	Yellow	↑14.00	↓2.40	↓2.70	↑13.00	↓12.20	↓2.90	↓19.00	↓14.40	↓4.00
	Blue	↓5.00	↑10.80	↑8.10	↑5.00	↓8.20	↓4.10	↓3.00	↓7.20	↓4.70
CIE L*u*v*	Red	↑8.00	↓4.60	↑2.10	↓20.00	↑8.60	↑14.40	↓14.00	↑6.00	↑10.70
	Yellow	↑14.00	↓2.40	↓2.70	↑13.00	↓12.20	↓2.90	↓19.00	↓14.40	↓4.00
	Blue	↓5.00	↑10.80	↑8.10	↑5.00	↓8.20	↓4.10	↓3.00	↓7.20	↓4.70
ANLAB	Red	↑8.00	↓4.60	↑2.10	↓20.00	↑8.60	↑14.40	↓14.00	↑6.00	↑10.70
	Yellow	↑14.00	↓2.40	↓2.70	↑13.00	↓12.20	↓2.90	↓19.00	↓14.40	↓4.00
	Blue	↓5.00	↑10.80	↑8.10	↑5.00	↓8.20	↓4.10	↓3.00	↓7.20	↓4.70
Hunter lab	Red	↑8.00	↓4.60	↑2.10	↓20.00	↑8.60	↑14.40	↓14.00	↑6.00	↑10.70
	Yellow	↑14.00	↓2.40	↓2.70	↑13.00	↓12.20	↓2.90	↓19.00	↓14.40	↓4.00
	Blue	↓5.00	↑10.80	↑8.10	↑5.00	↓8.20	↓4.10	↓3.00	↓7.20	↓4.70
FMC2	Red	↑8.00	↓4.60	↑2.10	↓20.00	↑8.60	↑14.40	↓14.00	↑6.00	↑10.70
	Yellow	↑14.00	↓2.40	↓2.70	↑13.00	↓12.20	↓2.90	↓19.00	↓14.40	↓4.00
	Blue	↓5.00	↑10.80	↑8.10	↑5.00	↓8.20	↓4.10	↓3.00	↓7.20	↓4.70
JPC79	Red	↑8.00	↓4.60	↑2.10	↓20.00	↑8.60	↑14.40	↓14.00	↑6.00	↑10.70
	Yellow	↑14.00	↓2.40	↓2.70	↑13.00	↓12.20	↓2.90	↓19.00	↓14.40	↓4.00
	Blue	↓5.00	↑10.80	↑8.10	↑5.00	↓8.20	↓4.10	↓3.00	↓7.20	↓4.70
CMC 1.0	Red	↑8.00	↓4.60	↑2.10	↓20.00	↑8.60	↑14.40	↓14.00	↑6.00	↑10.70
	Yellow	↑14.00	↓2.40	↓2.70	↑13.00	↓12.20	↓2.90	↓19.00	↓14.40	↓4.00
	Blue	↓5.00	↑10.80	↑8.10	↑5.00	↓8.20	↓4.10	↓3.00	↓7.20	↓4.70
BFD 1.0	Red	↑8.00	↓4.60	↑2.10	↓20.00	↑8.60	↑14.40	↓14.00	↑6.00	↑10.70
	Yellow	↑14.00	↓2.40	↓2.70	↑13.00	↓12.20	↓2.90	↓19.00	↓14.40	↓4.00
	Blue	↓5.00	↑10.80	↑8.10	↑5.00	↓8.20	↓4.10	↓3.00	↓7.20	↓4.70
CIE94 1.0	Red	↑8.00	↓4.60	↑2.10	↓20.00	↑8.60	↑14.40	↓14.00	↑6.00	↑10.70
	Yellow	↑14.00	↓2.40	↓2.70	↑13.00	↓12.20	↓2.90	↓19.00	↓14.40	↓4.00
	Blue	↓5.00	↑10.80	↑8.10	↑5.00	↓8.20	↓4.10	↓3.00	↓7.20	↓4.70

Remarks: ↑: higher than expected; ↓: lower than expected.

**Table 11:** RUI and visual levelness evaluation of dyed wool samples.

Sample	Water		Octane		Nonane	
	RUI	Visual	RUI	Visual	RUI	Visual
Red 0.1%	0.40	Good	0.45	Good	0.26	Good
Red 0.5%	0.24	Good	0.48	Good	0.20	Good
Red 1.5%	0.13	Excellent	0.20	Good	0.43	Good
Red 2.5%	0.14	Excellent	0.13	Excellent	0.14	Excellent
Red 3.5%	0.09	Excellent	0.44	Excellent	0.30	Good
Yellow 0.1%	0.09	Excellent	0.04	Excellent	0.05	Excellent
Yellow 0.5%	0.06	Excellent	0.05	Excellent	0.12	Excellent
Yellow 1.5%	0.10	Excellent	0.13	Excellent	0.04	Excellent
Yellow 2.5%	0.09	Excellent	0.15	Excellent	0.08	Excellent
Yellow 3.5%	0.05	Excellent	0.13	Excellent	0.05	Excellent
Blue 0.1%	0.22	Good	0.12	Excellent	0.31	Good
Blue 0.5%	0.16	Excellent	0.45	Good	0.42	Good
Blue 1.5%	0.46	Good	0.17	Excellent	0.11	Excellent
Blue 2.5%	0.08	Excellent	0.14	Excellent	0.34	Good
Blue 3.5%	0.08	Excellent	0.07	Excellent	0.08	Excellent
Mixture 0.3%	0.19	Excellent	0.28	Excellent	0.20	Good
Mixture 1.5%	0.34	Good	0.22	Excellent	0.29	Good
Mixture 3.0%	0.08	Excellent	0.14	Excellent	0.18	Excellent
